# Smartphone Addiction Modulates Object-Based Attention

**DOI:** 10.3390/bs16091581

**Published:** 2026-09-07

**Authors:** Jiulin Liu, Tianyi Yang, Yunfei Gao, Yi Zhang, Feng Kong, Jingjing Zhao

**Affiliations:** 1 School of Psychology, Shaanxi Normal University, Xi’an 710062, China; jiulinliu@snnu.edu.cn (J.L.); 13871939271@163.com (T.Y.); gyyunfei@126.com (Y.G.); zhangyi3036@snnu.edu.cn (Y.Z.); 2 Shaanxi Provincial Key Laboratory of Behavior and Cognitive Neuroscience, Xi’an 710062, China

**Keywords:** smartphone addiction, object-based attention, attentional shifting

## Abstract

Smartphone addiction has been linked to altered attentional functioning. However, it remains unclear whether these alterations are associated with enhanced attentional bias or impaired attentional shifting. Moreover, previous studies have largely relied on self-report measures, and evidence for attentional bias has mainly been obtained using smartphone-related stimuli. This study employed the classic two-rectangle paradigm to compare selective attention (including space-based and object-based attention) between individuals with high and low levels of smartphone addiction and to determine whether smartphone addiction primarily relates to attentional bias or attentional shifting. The results indicated that smartphone addiction significantly modulated object-based attention. More precisely, the high-addiction group exhibited a larger object-based attention effect than the low-addiction group, a difference primarily driven by the significantly longer reaction times of high-addiction individuals in the invalid different-object (IDO) condition, while no group difference was observed in the invalid same-object (ISO) condition. Furthermore, the high-addiction group exhibited significantly slower overall reaction times in the experimental tasks. These findings suggest that smartphone addiction is associated with attentional functioning and may involve poorer attentional shifting performance during object-based attention.

## 1. Introduction

While the convenience of smartphones has revolutionized information acquisition, social interaction, and entertainment, various user- and system-related factors may further encourage users to adopt and engage with mobile applications (Addula, 2025). Against this backdrop, the issue of addiction resulting from over reliance has become increasingly prominent. Smartphone addiction, often termed problematic smartphone use, refers to a maladaptive pattern of excessive and poorly regulated smartphone use that persists despite adverse consequences (Bianchi & Phillips, 2005; Hong et al., 2020; Kwon et al., 2013; Liang et al., 2026). It should be noted that the construct of “smartphone addiction” remains conceptually contested in the literature. Some scholars argue that the term “addiction” implies a clinical syndrome comparable to substance dependence, for which the evidence is currently insufficient (Billieux, 2012; Larsen et al., 2023; Panova & Carbonell, 2018). Consequently, alternative labels such as “problematic smartphone use” have been proposed to describe the same behavioral pattern without presuming a clinical disorder (Billieux et al., 2015; Nawaz, 2024). In the present study, following the convention of prior behavioral research (e.g., Liang et al., 2026), we use smartphone addiction to align with the terminology of the Smartphone Addiction Scale (SAS).

Extensive research has demonstrated that smartphone addiction is negatively associated with psychological well-being, academic performance, and sleep quality (Bjornsen & Archer, 2015; Demirci et al., 2015; Elhai et al., 2019; Fekih-Romdhane et al., 2022; Hartanto & Yang, 2016; Lepp et al., 2014; R.-D. Liu et al., 2019; Samaha & Hawi, 2016). However, few studies have examined the relationship between smartphone addiction and early cognitive processing, especially its core mechanism—selective attention. According to the filter model (Broadbent, 1958), attentional selection is the first “bottleneck” in information processing. Due to the limited capacity of brain information processing, individuals must rely on selective attention to effectively process a small portion of information (Posner & Petersen, 1990). If smartphone addiction affects information processing at this stage, then the subsequent memory, inhibitory control, and executive function (Farchakh et al., 2020; Frost et al., 2019; Hadlington, 2015; Hong et al., 2020; Jiang et al., 2023; Liang et al., 2026; Ndayambaje & Okereke, 2025; Wilmer et al., 2017) will inevitably be based on flawed original data. Therefore, analyzing associations between smartphone addiction and attentional function is critical for uncovering the underlying mechanisms of its associated cognitive impairments (Chun et al., 2011; Posner & Petersen, 1990).

Furthermore, existing research on the association between smartphone addiction and attention has primarily focused on attentional control and the functioning of attentional networks (Liang et al., 2026; Wilmer et al., 2017), and these studies have predominantly relied on self-report measures to assess attention (Hadar et al., 2017; Kobayashi et al., 2025; H. Zhao & Shapka, 2026). There remains a scarcity of research investigating the association between smartphone addiction and selective attention, particularly object-based selective attention. Although studies have found that smartphone-addicted individuals exhibit significant attentional bias and prolonged maintenance toward “smartphone-related stimuli”, such as mobile devices and app icons (D. Liu et al., 2025; D. Liu & Yang, 2024; Zhang et al., 2024), these phenomena primarily reflect domain-specific object-based attention driven by motivation or reward. Consequently, they mirror experience-dependent processing of specific stimuli rather than general selective attention mechanisms. Therefore, an important, yet unanswered question is whether the association between smartphone addiction and selective attention is limited to the specific stimuli associated with smartphones or can it generalize to more general objects? On the other hand, previous studies have also found that smartphone addiction is associated with impairments in attentional shifting and switching (Kobayashi et al., 2025; Zhang et al., 2024). For example, Kobayashi et al. (2025) found that problematic smartphone users exhibited increased volume of the right nucleus accumbens (NAcc), and this structural alteration was significantly associated with difficulties in attentional switching.

Existing findings point to two plausible accounts for attentional abnormalities linked to smartphone addiction: enhanced attentional bias and impaired attentional shifting. Yet, these two mechanisms have not been directly contrasted within a unified selective attention framework. It therefore remains unclear whether associations of smartphone addiction with selective attention primarily stem from enhanced attentional bias toward objects or from difficulties in shifting attention between them.

Selective attention refers to the process of prioritizing the processing of information relevant to the current task while simultaneously inhibiting irrelevant information (Chun et al., 2011; Moore & Zirnsak, 2017; Panichello & Buschman, 2021). Previous research has confirmed that selective attention can be deployed not only in space (space-based attention, stimuli within a specific spatial location are prioritized) (Eriksen & St. James, 1986; Müller & Kleinschmidt, 2003), but also be allocated within objects (object-based attention, features belonging to the same object are easier to detect than those belonging to different objects) (Duncan, 1984; Egly et al., 1994). The two-rectangle paradigm proposed by Egly et al. (1994) is used to examine both space-based and object-based attention at the same time. The task began with the presentation of a central fixation point and two rectangles positioned either horizontally or vertically. One end of a rectangle was then highlighted by a cue. After a brief delay, the target may appear in one of three positions: the cued position (valid trial), another position within the same rectangle (invalid same-object trial), or an equidistant position in the other rectangle (invalid different-object trial). Results typically show that reaction times are fastest for valid trials compared to invalid trials, reflecting space-based attention. The object-based attention effect emerges under invalid conditions, manifested as significantly reduced response latencies in invalid same-object (ISO) compared to invalid different-object (IDO) trials (Diao et al., 2024; Song et al., 2021; J. Zhao et al., 2020).

Among the theoretical accounts of object-based attention, two are particularly relevant to the present study. According to the attentional spreading theory (or sensory enhancement theory), attentional resources are driven to spontaneously spread across the contours of the cued object, and this spread enhances the perceptual representation of the object, thereby increasing the attentional bias toward the cued object and facilitating the processing of locations within it. Accordingly, object-based attention is attributed to enhanced processing efficiency in the ISO condition (Z. Chen & Cave, 2006; Richard et al., 2008). In contrast, the attentional shifting account proposes that object-based attention arises from the additional cost of shifting attention between distinct objects, resulting in slower responses in the IDO condition (Brown & Denney, 2007; Lamy & Egeth, 2002).

Building on previous theoretical and empirical work, this study aims to assess smartphone addiction using the Chinese revised version of the Smartphone Addiction Scale (SAS), originally developed by Kwon et al. (2013), and systematically examine the association between smartphone addiction and selective attention, particularly object-based attention and its underlying mechanisms, through the two-rectangle paradigm. Building upon existing research, we propose the following hypotheses: First, given that smartphone addiction is associated with attentional fragmentation, impaired alertness and sustained attention in daily life, individuals with high smartphone addiction are expected to demonstrate deficits in basic attentional response (Hadar et al., 2017; Marty-Dugas et al., 2018). Therefore, we hypothesize that the high smartphone addiction group will exhibit significantly longer overall reaction times during the experimental tasks compared to the low addiction group. Second, individuals with stronger attentional control tend to exhibit a smaller object-based attention effect (Hu et al., 2022). According to Attentional Control Theory, this relationship may arise because efficient attentional control reduces interference from irrelevant information while enabling attentional resources to be allocated in a goal-directed manner (Berggren & Derakshan, 2013; Eysenck & Derakshan, 2011). Given that the attentional control and interference-resistance abilities of smartphone-addicted individuals are impaired, we hypothesize that the object-based attention effect observed in the high smartphone addiction group will be significantly larger than that in the low addiction group. Finally, although a larger object-based attention effect is expected in the high smartphone addiction group, the cognitive process underlying this difference remains unclear. Based on the two theoretical accounts described above, two possible predictions can be made. If the attentional bias toward smartphone-related stimuli generalizes to neutral objects, this generalized bias may be expressed as enhanced attentional spreading within the cued object. Consequently, the high smartphone addiction group should exhibit shorter reaction times in the ISO condition than the low smartphone addiction group. Alternatively, if the high smartphone addiction group is associated with poorer attentional shifting, we propose that the larger object-based attention in the high-addiction group stems primarily from difficulties in attentional shifting; specifically, their reaction times in the IDO condition should be prolonged.

## 2. Method

### 2.1. Participants

The requisite sample size was determined using MorePower 6.0 (Campbell & Thompson, 2012). For a two-way mixed-design ANOVA with a significance level of α = 0.05, a statistical power of (1 − β) = 0.8, and an effect size of f = 0.07, MorePower indicated that a minimum of 108 participants was required. Based on this analysis, we recruited 213 undergraduate students from Shaanxi Normal University to complete the SAS. The sample consisted of 50 males and 163 females, with a mean age of 19.54 years (SD = 1.43). Participants with scores in the top and bottom 27% were allocated to the high and low smartphone addiction groups, respectively, with 57 participants in each group, and completed the two-rectangle cueing paradigm. This extreme-group selection strategy was adopted to ensure clear differentiation in smartphone addiction levels between groups and optimize experimental efficiency. All recruited participants provided written informed consent before the study in exchange for monetary compensation. The experimental protocol was approved by the Ethics Committee of Shaanxi Normal University.

### 2.2. Apparatus and Stimuli

In this study, the SAS was used to assess the level of smartphone addiction among participants. The scale consists of 7 items, using a 6-point Likert scale (from “strongly disagree” to “strongly agree”). Cronbach’s alpha coefficient for the scale was 0.80. Notably, one of the items was an attention-check question, designed to ensure that participants completed the questionnaire conscientiously.

Utilizing a two-rectangle cueing paradigm to assess selective attention, visual elements were displayed via a 19-inch screen at 1920 × 1080 resolution with a 60 Hz refresh rate, featuring a pair of parallel white rectangular configurations. Each rectangle measured 1.3° × 4.5°, with a 1.8° separation between them, and a central fixation cross (0.3° × 0.3°). A red cue (0.2°) appeared around one of the four ends of the rectangles. The target was a white letter “T” measuring 0.6° × 0.6°.

### 2.3. Design and Procedure

The study employed a 2 (Smartphone Addiction: High, Low) × 3 (Cue Validity: Valid, Invalid same-object, Invalid different-object) mixed-design, with smartphone addiction as a between-subject variable and cue validity as a within-subject variable. The dependent variable was reaction time (RT).

**Figure 1:**
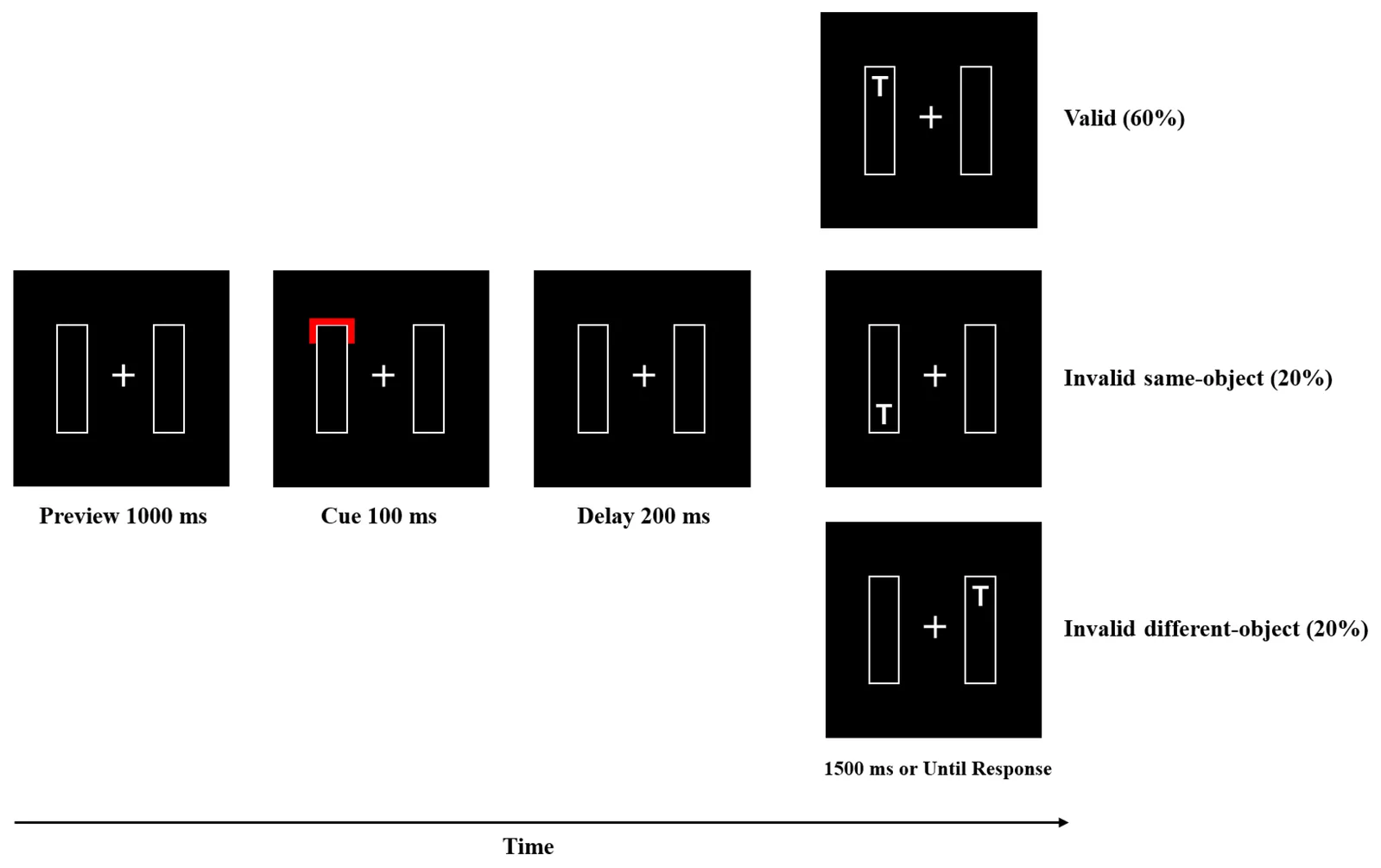
Experimental procedure of the two-rectangle cueing paradigm.

As depicted in Figure 1, each trial began with a 1000 ms exposure to a black background displaying a central fixation cross alongside two rectangles, followed by a 100 ms cue presented at one of the four rectangle ends. After a 200 ms stimulus onset asynchrony (SOA), the target letter “T” appeared at one of the four rectangle ends and remained visible until a response was made. Participants were instructed to maintain fixation on the center and press the “M” key as quickly and accurately as possible upon detecting the target. The cues appeared with equal probability at one of the four ends of the two rectangles. The target appeared in three conditions: 60% of trials at the cued location (Valid), 20% of trials at the uncued end of the cued rectangle (ISO), and 20% of trials at the equidistant end of the uncued rectangle (IDO). The experiment comprised 20 practice trials and 384 experimental trials (4 blocks of 96 trials each), lasting approximately 20 min, including 64 catch trials to maintain alertness.

### 2.4. Data Analysis

All statistical analyses were performed using JASP software (Version 0.96). For each condition, trials with reaction times exceeding three standard deviations from the participant’s mean were excluded from the analysis; this procedure resulted in the removal of 3.6% of the total trials. Prior to conducting the ANOVAs, the normality of the response time data was assessed using the Shapiro–Wilk test, and the homogeneity of variance was examined using Levene’s test. No substantial violations of the normality or homogeneity-of-variance assumptions were observed. We therefore conducted two-way mixed-design ANOVAs on RT separately for the space-based and object-based effects.

## 3. Results

### 3.1. Group Validation (SAS)

An independent-samples *t*-test was conducted to compare smartphone addiction scores between the high-addiction and low-addiction groups. The results showed that the scores of the high-addiction group (M = 29.75, SD = 2.38) were significantly higher than those of the low-addiction group (M = 18.11, SD = 2.76), t(112) = 24.13, *p* < 0.001, Cohen’s d = 4.52, confirming the validity of our grouping procedure.

### 3.2. Space-Based Effects (SBA)

**Figure 2:**
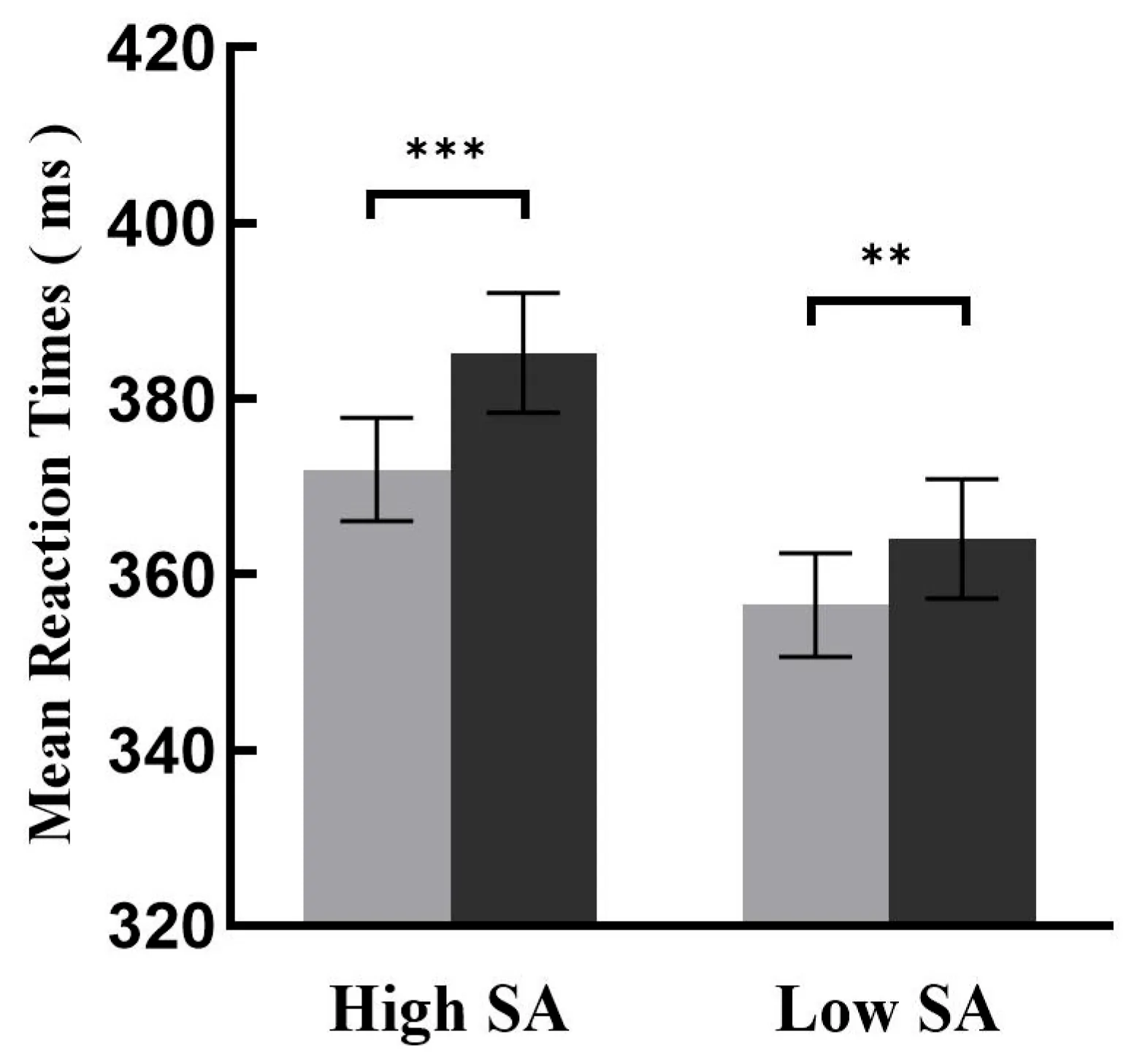
Interaction plot of spatial effect and addiction level. High SA represents the high smartphone addiction group, and Low SA represents the low smartphone addiction group. Grey color indicates the valid condition, whereas dark color indicates the invalid condition. *** *p* < 0.001; ** *p* < 0.01.

The space-based attention (SBA) effect was determined by the difference in RT between the valid and invalid conditions (SBA = RTinvalid − RTvalid). Invalid RT was calculated as the mean of the RTs in the ISO and IDO conditions. We performed a 2 (Cue Validity: Valid vs. Invalid) × 2 (Smartphone Addiction: High vs. Low) mixed-design ANOVA on the reaction times. The results are presented in Figure 2. The main effect of cue validity was significant, F(1, 112) = 37.27, *p* < 0.001, ηp2 = 0.25. Participants responded significantly faster in the valid condition (M = 364.22 ms, SE = 4.22 ms) than in the invalid condition (M = 374.64 ms, SE = 4.88 ms). The main effect of smartphone addiction was also significant, F(1, 112) = 4.29, *p* = 0.041, ηp2 = 0.037. Participants with high smartphone addiction exhibited significantly slower responses (M = 378.55 ms, SE = 4.56 ms) than those with low smartphone addiction (M = 360.25 ms, SE = 4.45 ms). The interaction between cue validity and smartphone addiction was not significant, F(1, 112) = 2.84, *p* = 0.095, ηp2 = 0.025. These results indicate that smartphone addiction does not significantly modulate spatial attention.

Given that the interaction between cue validity and smartphone addiction yielded a *p*-value of 0.095, suggesting a trend toward significance, we further assessed the evidence for this interaction by conducting a Bayesian ANOVA on the Space-Based Effects. The results are presented in Table 1. The best-performing model included cue validity and smartphone addiction but excluded their interaction. This model received a higher posterior model probability (P(M|data) = 0.43) than the corresponding model including the interaction (P(M|data) = 0.25). Using the model without the interaction as the reference model (BF_10_ = 1), the model including the interaction had a relative Bayes factor of 0.59. This corresponds to BF_01_ = 1.69, providing weak evidence in favor of the model without the interaction.

### 3.3. Object-Based Effects (OBA)

**Figure 3:**
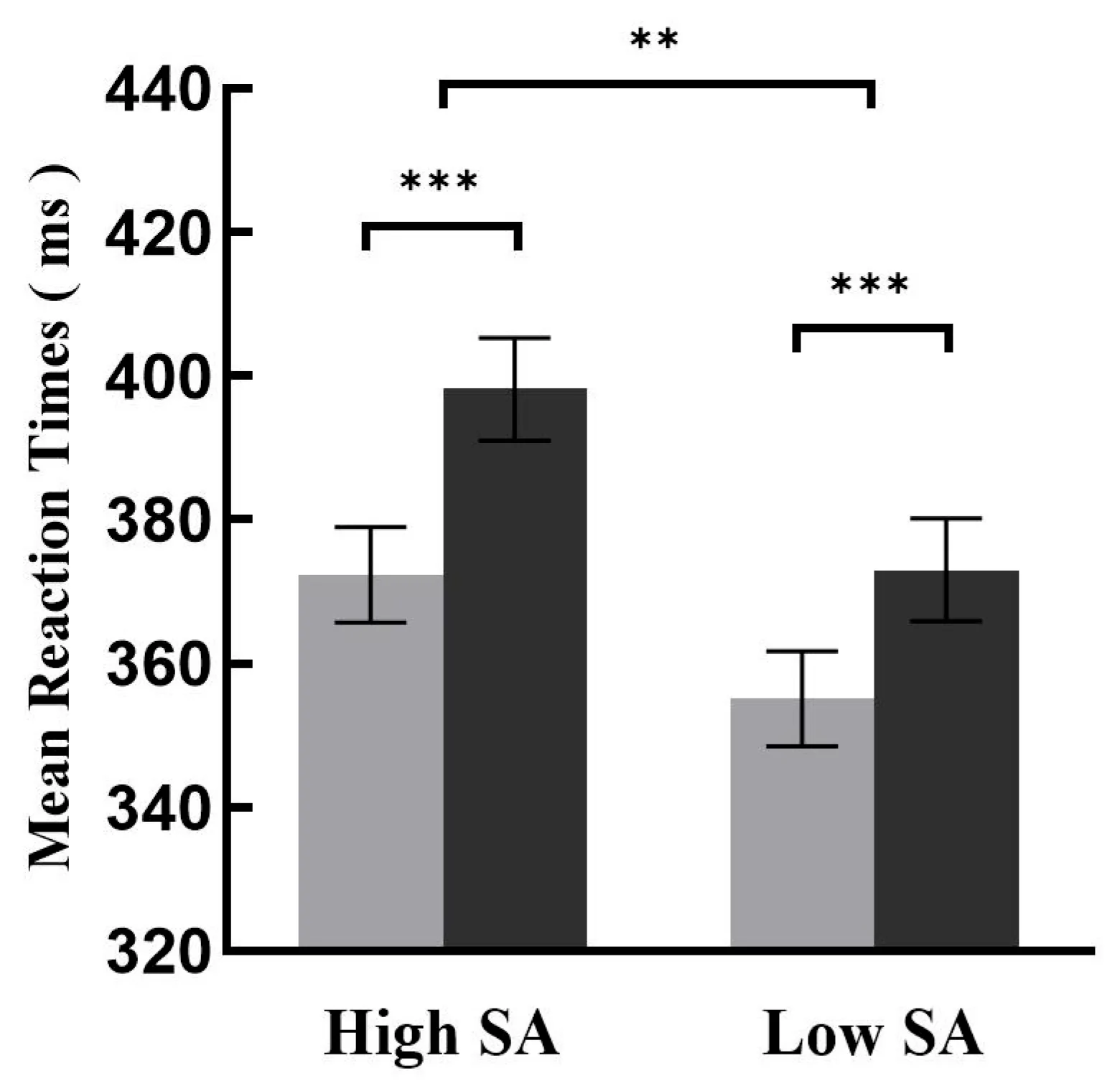
Interaction plot of object effect and addiction level. High SA represents the high smartphone addiction group, and Low SA represents the low smartphone addiction group. Grey color indicates the ISO condition, whereas dark color indicates the IDO condition. *** *p* < 0.001; ** *p* < 0.01.

The object-based effect was calculated based on the difference in RT between ISO and IDO conditions. We conducted a two-way mixed-measures ANOVA on RT, with cue validity (ISO vs. IDO) as a within-subject factor and smartphone addiction level (High vs. Low) as a between-subject factor. The results are presented in Figure 3. The main effect of cue validity was significant, F(1, 112) = 252.37, *p* < 0.001, ηp2 = 0.693. Participants responded significantly faster in the ISO condition (M = 363.70 ms, SE = 4.71 ms) than in the IDO condition (M = 385.58 ms, SE = 5.15 ms). The main effect of smartphone addiction was also significant, F(1, 112) = 4.86, *p* = 0.03, ηp2 = 0.042. Participants with high smartphone addiction exhibited significantly slower responses (M = 385.23 ms, SE = 5.21 ms) than those with low smartphone addiction (M = 364.05 ms, SE = 4.66 ms). Critically, the interaction between cue validity and smartphone addiction was significant, F(1, 112) = 8.29, *p* = 0.005, ηp2 = 0.069. Simple effect analysis indicated that the object-based effect was larger in the high-addiction group (25.84 ms, SE = 2.04 ms) than that in the low-addiction group (17.91 ms, SE = 1.85 ms), and this difference stemmed from the slower RTs in the IDO condition of the high-addiction group compared to that of the low-addiction group, t(112) = 2.497, *p* = 0.04, Cohen’s d = 0.479.

These results suggest that smartphone addiction modulates object-based attention, with the significant interaction primarily driven by the slower RTs observed in the high-addiction group under the IDO condition, whereas no significant between-group difference was observed under the ISO condition. This pattern suggests that the two groups did not differ in attentional spreading within the same object, whereas the high-addiction group showed response slowing when attention had to be shifted between different objects. However, this finding should be interpreted with caution, as the generally slower responses observed in the high-addiction group may have contributed to this pattern. For further details, please refer to Table 2.

We also conducted a Bayesian ANOVA on the Object-Based Effects. The results are presented in Table 3. The best-performing model included cue validity, smartphone addiction, and their interaction, and received the highest posterior model probability (P(M|data) = 0.77). By contrast, the model including the two main effects only (cue validity + smartphone addiction, no interaction) obtained a substantially lower posterior model probability (P(M|data) = 0.12). Using the model including the interaction as the reference model (BF_10_ = 1), the model excluding the interaction had a relative Bayes factor of 0.16. This corresponds to BF_10_ = 6.25 in favor of the model including the interaction. Thus, the results provided evidence favoring the model including the cue validity × smartphone addiction interaction.

## 4. Discussion

In this study, we employed the classic two-rectangle cueing paradigm to investigate associations between smartphone addiction and individuals’ space-based and object-based attention. The experimental results validated the efficacy of the paradigm: all participants exhibited significant space-based and object-based attentional effects. Our core finding demonstrated that smartphone addiction significantly modulated object-based attention: participants in the high-addiction group exhibited a significantly stronger object-based effect than those in the low-addiction group. Further analysis revealed that this difference primarily stemmed from significantly longer reaction times in the high-addiction group during the IDO condition compared to the low-addiction group. Concurrently, the high-addiction group exhibited a slower overall RT compared to the low-addiction group.

Regarding space-based attentional effects, valid cues effectively oriented spatial attention, yielding markedly reduced reaction times compared to invalid trials. This result is consistent with the classic spatial cueing effect proposed by Posner and Petersen (1990), reflecting the ability of spatial cues to effectively guide the allocation of attentional resources to target locations, thereby enhancing information processing efficiency (Diao et al., 2024; Song et al., 2021; J. Zhao et al., 2020). Data also indicated that high-addiction individuals exhibited significantly slower overall RTs compared to low-addiction individuals. This is consistent not only with existing research suggesting that smartphone addiction leads to a decline in cognitive processing efficiency (B. Chen et al., 2017; Hadlington, 2015) but also with the findings of Ralph et al. (2014), who observed that high media multitaskers demonstrated longer RTs in sustained attention tasks, collectively suggesting that smartphone addiction might impact attentional processing efficiency. Furthermore, we found no sufficient evidence that smartphone addiction modulated spatial attention, likely because spatial attention is a relatively stable attentional mechanism, consistent with previous research indicating that other individual difference factors, such as personality traits and cognitive styles, do not exert a moderating effect on spatial attention (Hu et al., 2020, 2022). Nevertheless, the *p*-value of 0.095 suggests a potential trend toward an interaction, whereas the Bayesian analysis provided only weak evidence favoring the model without the interaction. Therefore, the current evidence is insufficient to draw a definitive conclusion regarding whether smartphone addiction modulates spatial attention, and future studies with larger samples are needed to further clarify this issue.

In our analysis of object-based attentional effects, a significantly larger object-based effect was observed in the high-addiction group than in the low-addiction group. Previous research has confirmed that weak attentional control capacity is associated with a stronger object-based attention effect (Hu et al., 2022). This suggests that the attentional control capacity of individuals with smartphone addiction is impaired (Hong et al., 2020; Liang et al., 2026; Marty-Dugas et al., 2018; H. Zhao & Shapka, 2026). This result is consistent with B. Chen et al. (2017), who found that excessive smartphone users exhibited a significant decline in control capacity. High-addiction individuals showed slower RTs in the IDO condition than low-addiction individuals, with no significant difference in the ISO condition. This pattern provides a critical entry point for understanding the attentional mechanisms underlying smartphone addiction. According to the attentional shifting hypothesis, the IDO condition primarily reflects the process of attentional shifting (Brown & Denney, 2007; Lamy & Egeth, 2002). Therefore, the larger between-group difference observed in the IDO condition is consistent with the possibility that individuals with high smartphone addiction experience relatively greater difficulty when attentional processing requires shifts between objects. However, this interpretation should be treated with caution because the high-addiction group also exhibited generally slower responses. This interpretation is broadly consistent with the findings of Kobayashi et al. (2025), who observed a significant positive correlation between problematic smartphone use and reduced attentional switching ability. This observation also aligns with evidence from the media multitasking literature, where chronic multitasking not only fails to enhance switching performance but conversely leads to poorer task accuracy and higher attentional switch costs (Uncapher & Wagner, 2018). The mechanism underlying the ISO condition is typically associated with attentional bias and the process of attentional spreading within an object. Although smartphone-addicted individuals are more easily attracted by stimuli such as mobile devices (D. Liu et al., 2025; D. Liu & Yang, 2024; Zhang et al., 2024), this study did not find a significant difference in the ISO condition, suggesting that smartphone addiction is not associated with a generalized attentional bias toward neutral cued objects. This finding suggests that such attentional bias was stimulus-specific rather than reflecting a generalized enhancement of attentional bias. From the perspective of attentional spreading theory (Z. Chen & Cave, 2006; Shomstein & Yantis, 2002), there is no significant difference in attentional spreading capacity within a neutral object between high and low smartphone-addicted individuals. Therefore, future research could combine smartphone image stimuli with the two-rectangle paradigm to further investigate the relationship between smartphone addiction and attentional mechanisms, expecting to observe faster RTs in the ISO condition in individuals with high smartphone addiction (Haruki et al., 2025; D. Liu et al., 2025; D. Liu & Yang, 2024; Zhang et al., 2024). It should be noted that the longer reaction times observed in the high-addiction group in the IDO condition cannot be attributed solely to poorer attentional shifting ability. This pattern may also be associated with generally slower responding among high-addiction participants. The present findings, however, cannot determine whether a specific difficulty in shifting attention between objects contributes to the generally slower responding observed in the high-addiction group, or whether generally slower responding becomes particularly pronounced when attentional shifting between objects is required. Taken together, although the interaction pattern is consistent with a relative difficulty in attentional shifting among individuals with high smartphone addiction, the contribution of general response slowing to the observed effect cannot be ruled out.

Some constraints of this work warrant consideration in subsequent investigations. First, although we employed an extreme group design by comparing the top 27% and bottom 27% of participants on the smartphone addiction scale, the current undergraduate population generally exhibits a high tendency for smartphone reliance. The sample data showed a skewed distribution toward the higher score range, which to some extent weakened the differences in attentional performance across different levels of addiction. Meanwhile, this selection strategy meant that our findings cannot be generalized to individuals with intermediate levels of smartphone addiction. Furthermore, we did not fully utilize information from the continuous measure, further limiting generalizability across the continuum of smartphone addiction severity (Fisher et al., 2020; Preacher et al., 2005). Future studies should recruit participants across a broader range of smartphone addiction levels and examine smartphone addiction as a continuous individual difference variable. Second, another limitation concerns our reliance on self-reported smartphone addiction scores. Although the SAS captures important characteristics of problematic smartphone use, self-reported assessments may not accurately reflect objectively measured smartphone use or screen time and may be influenced by recall errors and subjective perceptions. Thus, the present study cannot determine whether the observed attentional differences are associated with perceived problematic smartphone use or actual smartphone use. Future research should combine self-report measures with objective smartphone-use monitoring, such as device-recorded screen time and application-level usage data, to provide a more comprehensive assessment of smartphone use. Third, this study employed a cross-sectional design, which precludes inferences regarding causality between smartphone addiction and attentional function. Theoretically, smartphone addiction may lead to impaired attentional function, but individuals with poorer attentional function may also be more susceptible to developing smartphone dependency. To definitively establish causality, upcoming investigations are encouraged to employ either longitudinal methods or controlled interventions. Finally, the present study focused primarily on individual-level attentional mechanisms and did not consider technology- and app-related design features, such as push notifications and endless scrolling, that may influence attentional processing. Future research should integrate individual cognitive characteristics with technology-related design features to provide a more comprehensive and ecologically valid understanding of the relationship between smartphone addiction and attentional function.

## 5. Conclusions

Using the classic two-rectangle cueing paradigm, we found that participants with high smartphone addiction showed a stronger object-based effect, primarily driven by prolonged RTs under the IDO condition. Additionally, these individuals also displayed generally slower overall responses. Collectively, the present study provides evidence that smartphone addiction is associated with attentional functioning and may involve poorer attentional shifting performance during object-based attention.

## Figures and Tables

**Table 1:** Bayesian Model Comparisons for Space-Based Effects.

	P(M)	P(M|data)	BF_10_	Error
CV + SA	0.20	0.43	1.00	
CV	0.20	0.32	0.74	7.59
CV + SA + CV × SA	0.20	0.25	0.59	8.86
SA	0.20	1.05 × 10^−6^	2.44 × 10^−6^	12.99
Null model	0.20	6.18 × 10^−7^	1.44 × 10^−6^	7.54

Note: P(M) and P(M|data) indicate prior and posterior model probabilities, respectively. The first row shows the best-performing model. BF_10_ indicates Bayes factors relative to the best-performing model; and error is the relative error associated with the numerical method used to estimate the Bayes factors. CV = Cue Validity; SA = Smartphone Addiction.

**Table 2:** Mean RTs during the test phase of the experiment. The error terms, in parentheses, reflect the within-subjects SEM.

	Valid	Invalid	ISO	IDO
High SA	371.93 (5.63)	385.23 (7.12)	372.31 (6.82)	398.15 (7.55)
Low SA	356.50 (6.17)	364.05 (6.45)	355.09 (6.36)	373.00 (6.67)

**Table 3:** Bayesian Model Comparisons for Object-Based Effects.

	P(M)	P(M|data)	BF_10_	Error
CV + SA + CV × SA	0.20	0.77	1.00	
CV + SA	0.20	0.12	0.16	11.91
CV	0.20	0.11	0.14	8.15
SA	0.20	2.41 × 10^−27^	3.11 × 10^−27^	9.03
Null model	0.20	1.21 × 10^−27^	1.56 × 10^−27^	8.10

## Data Availability

Data will be made available with reasonable request to the corresponding author.
